# Recent Advances in Smart Farming

**DOI:** 10.3390/ani12060705

**Published:** 2022-03-11

**Authors:** Pedro Gonçalves, Paulo Pedreiras, António Monteiro

**Affiliations:** 1 Escola Superior de Tecnologia e Gestão de Águeda and Instituto de Telecomunicações, Campus Universitário de Santiago, Universidade de Aveiro, P3810-193 Aveiro, Portugal; 2 Departamento de Eletrónica, Telecomunicações e Informática and Instituto de Telecomunicações, Campus Universitário de Santiago, Universidade de Aveiro, P3810-193 Aveiro, Portugal; pbrp@ua.pt; 3 Agrarian School of Viseu, P3500-606 Viseu, Portugal; amonteiro@esav.ipv.pt

The Digital Transformation, which has the Internet of Things (IoT) as one of its pillars, is penetrating all aspects of our society with dramatic effects. In fact, buzzwords such as “Smart homes”, “Smart offices”, “Smart health” and “Smart factories”, to name just a few, have become a commonplace and reflect the profound structural changes that the Digital Transformation is having in the way citizens live their lives and how businesses and industries are organized.

Naturally, agriculture is not exempt the ongoing Digital Transformation, and “Smart farming”, along with similar terms such as “Precision Agriculture” and “Agriculture 4.0/5.0” eventually emerged to refer to the new management of farms made possible by the use of digital technologies, including advanced digital sensors, IoT, wireless sensor networks, artificial intelligence, cloud computing and autonomous robots. The progressive adoption of these technologies is making farming more industrialized and technology-driven, bringing a multitude of benefits such as increases in the quantity and quality of products, reduction in the human labor required and reductions in resource consumption. Furthermore, digital technologies are regarded as a keys to addressing actual and important societal challenges, such as adapting production in the face of climate change, increasing sustainability and increasing production levels in light of population growth. Therefore, it is expected that the relevance and penetration of these technologies will keep increasing steadily over the upcoming decades.

This Special Issue collects the latest research results and advances in technologies relevant to the “Smart Farming” domain. The collected papers address the use of sensor technologies combined with advanced algorithms, such as artificial intelligence, to monitor animals and gain knowledge about their behavior and welfare, as well as to automate and improve the efficiency of several aspects related with their management. “*Instance Segmentation with Mask R-CNN Applied to Loose-Housed Dairy Cows in a Multi-Camera Setting*” [1] proposes a system of eight surveillance cameras that record dairy cows, with the objective of automating cattle herd activity analysis. The system uses Convolutional Neural Networks to detect dairy cows in the video recordings, providing indications on how the number of self-annotated images reflects the performance of the model. “*Machine Learning-Based Microclimate Model for Indoor Air Temperature and Relative Humidity Prediction in a Swine Building*” [2] analyzes the performance of machine-learning models used for the prediction of indoor air temperature and indoor relative humidity in ventilated swine buildings, providing optimal input selection for their feeding. *“Design of Scalable IoT Architecture Based on AWS for Smart Livestock”* [3] presents a scalable cloud-based architecture targeting smart livestock monitoring, featuring services such as environmental monitoring, health, growth, reproduction, emotional state and stress levels of animals. “*SheepIT, an E-Shepherd System for Weed Control in Vineyards: Experimental Results and Lessons Learned*” [4] presents experimental results from a pilot test of a framework that not only collects behavior-related data with the objective of assessing the welfare of sheep, but also conditions its behavior, allowing sheep to be used, e.g., in vineyards, without presenting a risk to vines and fruits. “*Horse Jumping and Dressage Training Activity Detection Using Accelerometer Data*” [5] presents a system to help in the analysis and training of equestrian jumping and dressage movements. The system comprises accelerometer sensors whose data is fed to an advanced machine-learning algorithm to detect gaits, paces, jumps, flying changes and other features during jumping and dressage training. The system also provides relevant training metrics such as velocity, stride length and step frequency. “*Calf Birth Weight Predicted Remotely Using Automated in-Paddock Weighing Technology*” [6] proposes predictive models of calf birth weight from liveweight data collected remotely and individually via automated in-paddock walk-over-weighing scale. Results confirm that such models and in-paddock weighing systems can be used in conjunction to improve calving management and productivity. “*Influence of Subclinical Ketosis in Dairy Cows on Ingestive-Related Behaviours Registered with a Real-Time System*” [7] presents a study that uses pressure and acceleration sensors that detect and report, in real-time, chewing and head movement. These data are then processed to obtain cow behavioral data. Collected data allow the authors to conclude that cows with subclinical ketosis had reduced rumination time and rumination chews, drinking time, chews per minute, boluses, and chews per bolus, therefore it is shown that changes in rumination can be used for early identification of subclinical ketosis in dairy cows. Finally, “*A Novel Miniaturized Biosensor for Monitoring Atlantic Salmon Swimming Activity and Respiratory Frequency*” [8] addresses the use of a miniaturized biosensor to monitor individual swimming activity and metabolic condition of Atlantic salmon in land-based aquaculture systems. The results show that the impact of the tagging procedure is acceptable, and activity and behavior results are accurate.

This Special Issue also includes two review papers. “*Wearable Wireless Biosensor Technology for Monitoring Cattle: A Review*” [9] presents an interesting review of sensor systems available in the market, summarizing information on them and evaluating their accuracy. The paper concludes that most sensors have good performance in some aspects (e.g., eating time, ruminating time, etc.), but not in others (e.g., drinking time), identifying lines that deserve further R&D. On the other hand, “*Precision Agriculture for Crop and Livestock Farming—Brief Review*” [10] has a broader scope, reviewing scientific and technological trends in precision agriculture and their applications in crop and livestock farming, with the aim of serving as a guide, both for researchers and farmers, in the application of technology to agriculture.

The papers collected in this Special Issue reflect not only the significant advances that have been attained in many areas, but above all demonstrate that there is a vast and growing range of technologies that can be readily adopted in farming activities. They contribute significantly to improving efficiency, reducing environmental impacts and increasing productivity in this field, which is fundamental to our society.

## Data Availability

Not applicable.
